# Association between preterm births and socioeconomic development: analysis of national data

**DOI:** 10.1186/s12889-022-14376-2

**Published:** 2022-11-03

**Authors:** Marina Sanches Montemor, Gabriella Ferreira Demarque, Agatha Sacramento Rodrigues, Rossana Pulcinelli Vieira Francisco, Mario Henrique Burlacchini de Carvalho

**Affiliations:** 1 Faculdade de Medicina da Universidade de São Paulo, São Paulo, São Paulo Brasil; 2 Faculdade de Saúde Pública da Universidade de São Paulo, São Paulo, Brasil; 3 Departamento de Estatística, Universidade Federal Do Espírito Santo, Vitória, Espírito Santo, Brasil; 4 Departamento de Obstetrícia E Ginecologia, Disciplina de Obstetrícia, Faculdade de Medicina da Universidade de São Paulo, São Paulo, São Paulo Brasil

**Keywords:** Preterm birth, Socioeconomic status, Spontaneous preterm birth, Elective preterm birth

## Abstract

**Background:**

The increasing prevalence of preterm birth, which is a global phenomenon, is attributable to the increased medical indications, artificial gestations, and some socioeconomic factors. This study was conducted to identify whether development and equality indices are associated with the incidence of preterm birth, specifically, spontaneous and elective preterm births.

**Methods:**

This retrospective observational study comprised an analysis of data on live births from 2019 in Brazil and on socioeconomic indices that were derived from census information in 2017. Data were summarised using absolute and relative frequencies. Spearman’s correlation was used to determine the correlation between socioeconomic factors and the preterm birth rate. Multiple beta regression analysis was performed to determine the best model of socioeconomic covariates and preterm birth rate. The significance level was set at 5%.

**Results:**

In 2019 in Brazil, the preterm birth rate was 11.03%, of which 58% and 42% were spontaneous and elective deliveries, respectively. For all preterm births, Spearman’s correlation varied from *ρ* = 0.4 for the Gini Index and *ρ* =  − 0.24 for illiteracy. The best fit modelled the spontaneous preterm birth fraction as a negative function of the Human Development Index (HDI). The best-fit model considered the expected elective preterm birth fraction as a positive function of the HDI and as a negative function of the Gini Index, which was used as a precision parameter.

**Conclusions:**

We observed a reduction in the fraction of spontaneous preterm births; however, the distribution was not uniform in the territory: higher rates of spontaneous preterm birth were noticed in the north, northeast, and mid-west regions. Thus, areas with lower education levels and inequal income distribution have a higher proportion of spontaneous preterm birth. The fraction of elective preterm birth was positively associated with more advantaged indices of socioeconomic status.

**Supplementary Information:**

The online version contains supplementary material available at 10.1186/s12889-022-14376-2.

## Background

Preterm birth, which is defined as birth before 37 weeks of gestation, is a global phenomenon of increasing prevalence [1], which has also been observed in earlier Brazilian studies, including the Pelotas cohort wherein preterm births increased from 5.8% (1982) to 13.8% (2015) [2]. Global trends indicate that estimated prevalence of preterm births varies from 9.6% to 11.1%, and low- and middle-income countries are responsible for most of the burden [3, 4]. An increase in the preterm birth rates is attributable to both, an increase in medical indications and in artificial gestations [5], which have enhanced the rate of elective preterm births. Moreover, improved national information systems and advances in perinatal support are important factors for this increase and the higher life expectancy of children who are born preterm [1].

The mechanisms that lead to birth before 37 weeks of gestation are unclear and constitute an expanding field; however, three main factors for spontaneous preterm birth have been proposed: social stress and race; infection and inflammation; and genetics [6]. The consequences of preterm birth include higher perinatal morbimortality [5, 7] and an impact on child development and health as well as well-being in adulthood [3].

Most of the population-based studies that investigated the related factors and the incidence of preterm birth have been conducted in developed countries. In less developed countries, the relevant research tends to be observational and mostly single-centre studies, due to which the results are not representative of the general population [3].

Previous studies in the United States found that area poverty and deprivation, assessed by schooling, housing, occupation, race, and income indicators, are related to a higher risk of preterm birth [8, 9]. Furthermore, a meta-analysis that included 28 studies showed that lower-income neighbourhoods were associated with a higher risk of preterm birth [10]. A longitudinal study in Spain identified a higher risk of spontaneous preterm birth among pregnant women from countries that had a low or medium Human Development Index (HDI) [11].

Nonetheless, a Dutch study showed contradictory results: non-Western pregnant women who lived in neighbourhoods with a lower social index were not at a higher risk for adverse perinatal outcomes than those who lived in higher index locations [12]. Moreover, after adjusting for multiple comparisons, a study that was conducted in Hong Kong found no association between neighbourhood income, inequality, or the Gini Index and preterm birth [13].

This study aimed to contribute to this literature gap, identifying whether less-developed and equal areas of Brazil are associated with a higher incidence of preterm birth, specifically spontaneous and elective preterm births. The different socioeconomic factors associated with spontaneous and elective preterm births are not commonly explored features in contemporaneous literature.

## Methods

This retrospective observational study used nationwide data that were obtained from national database of live births, the Sistema de Informações sobre Nascidos Vivos (SINASC), and the Brazilian census conducted by the Brazilian Institute of Geography and Statistics (IBGE).

Preterm birth rates from 2014 to 2019 were analysed by the Brazilian Federal Unit (FU) and by year (Additional File 1). A pre-analysis showed that data from 2014 were more accurate due to recent improvements in preterm birth measures and notifications [14–16] and 2019 was the last year which had consolidated data; therefore, the interval from 2014 to 2019 were considered for analysis in this study and data from 2019 were selected for further analysis. Data were extracted from the Data Science Applied to Health Platform (PCDaS) [17] of the Oswaldo Cruz Foundation (Fiocruz*)*.

Census data were extracted from the Pesquisa Nacional por Amostra de Domicílio (PNAD) of 2017, and included HDI and its sub-components – life expectancy, education, and gross national income (GNI) per capita; the Gini Index; illiteracy; access to water; access to sanitation; and life expectancy of Brazilian FUs. Preliminary analysis showed no significant variation in the above-mentioned indices throughout the study period; accordingly, the most recent data from 2017 for all indices, except for life expectancy (for which the most recent data was from 2018), were used. Socioeconomic metrics were extracted from the Atlas of Human Development in Brazil (*Atlas Brasil*) [18] and from the Health System Performance Assessment Methodology (PROADESS) platform. The SINASC and IBGE census records contain publicly available anonymised data. Therefore, with regard to the Brazilian ethics regulatory requirements, no prior informed consent or ethical approval by the institutional review board were required [19].

Spontaneous delivery comprised all forms of non-induced labour except for elective caesarean birth, remaining only cases with spontaneous onset of labour. Elective delivery was defined as provider-initiated, which included induction of labour and elective caesarean birth (either because of maternal or foetal indications, or other non-medical reasons) [20].

The preterm birth rate was calculated as:

$$\mathrm{Preterm}\;\mathrm{birth}\;\mathrm{rate}\;=\;\mathrm{Number}\;\mathrm{of}\;\mathrm{births}\;\mathrm{from}\;22\;\mathrm{to}\;37\;\mathrm{weeks}\;\mathrm{of}\;\mathrm{gestation}\;/\;(\mathrm{total}\;\mathrm{number}\;\mathrm{of}\;\mathrm{births}\;-\;\mathrm{number}\;\mathrm{of}\;\mathrm{births}\;\mathrm{missing}\;\mathrm{for}\;\mathrm{gestational}\;\mathrm{age})$$

The spontaneous preterm birth rate was calculated as:

$$\mathrm{Spontaneous}\;\mathrm{preterm}\;\mathrm{birth}\;\mathrm{rate}\;=\;\mathrm{Number}\;\mathrm{of}\;\mathrm{births}\;\mathrm{with}\;\mathrm{spontaneous}\;\mathrm{delivery}\;\mathrm{from}\;22\;\mathrm{to}\;37\;\mathrm{weeks}\;\mathrm{of}\;\mathrm{gestation}\;/\;(\mathrm{total}\;\mathrm{number}\;\mathrm{of}\;\mathrm{births}\;-\;\mathrm{number}\;\mathrm{of}\;\mathrm{births}\;\mathrm{missing}\;\mathrm{for}\;\mathrm{gestational}\;\mathrm{age})$$

Similarly, the elective preterm birth rate was calculated as:

$$\mathrm{Elective}\;\mathrm{preterm}\;\mathrm{birth}\;\mathrm{rate}\;=\;\mathrm{Number}\;\mathrm{of}\;\mathrm{births}\;\mathrm{via}\;\mathrm{elective}\;\mathrm{delivery}\;\mathrm{from}\;22\;\mathrm{to}\;37\;\mathrm{weeks}\;\mathrm{of}\;\mathrm{gestation}\;/\;(\mathrm{total}\;\mathrm{number}\;\mathrm{of}\;\mathrm{births}\;-\;\mathrm{number}\;\mathrm{of}\;\mathrm{births}\;\mathrm{missing}\;\mathrm{for}\;\mathrm{gestational}\;\mathrm{age})$$

The fraction of spontaneous preterm births was defined as the number of births via spontaneous preterm delivery from 22 to 37 weeks of gestation divided by the total number of births from 22 to 37 weeks of gestation, and the fraction of elective preterm births was calculated as the number of births with elective delivery from 22 to 37 weeks of gestation divided by the total number of births from 22 to 37 weeks of gestation.

The data were summarised as absolute and relative (%) frequencies. Spearman’s correlation was used to determine the correlation between socioeconomic indicators and the preterm birth rate. Multiple analyses were performed using a beta regression model with a logit link function [21], because the preterm birth rate varies within the interval (0,1). As we have only 27 subjects (27 FUs) for use in the multiple regression model, we performed a pre-selection analysis, which ascertained whether the Spearman’s correlation between two socioeconomic variables was greater than 0.8. The factors with the highest correlation with the preterm birth rate were selected as covariates for inclusion in the beta regression model for preterm birth rate.

The likelihood ratio test for heteroskedasticity was performed to assess which of the following regression models was more suitable: fixed or variable dispersion beta regression (where Φ is the dispersion parameter) [22]. For preterm birth, specific regressions were performed for ascertaining the spontaneous and elective preterm birth fractions. Akaike Criteria [23] were used to determine the best model and diagnostic tools in beta regression were performed to evaluate model fit and assumptions [22]. The factors importance was presented in terms of permutation feature importance graph by considering the gain in terms of Mean Square Error (MSE) [24]. The significance level was established at 5%. All analyses were performed using R (https://www.r-project.org, version 4.0.3).

## Results

### Total number of births and preterm births

**Fig. 1:**
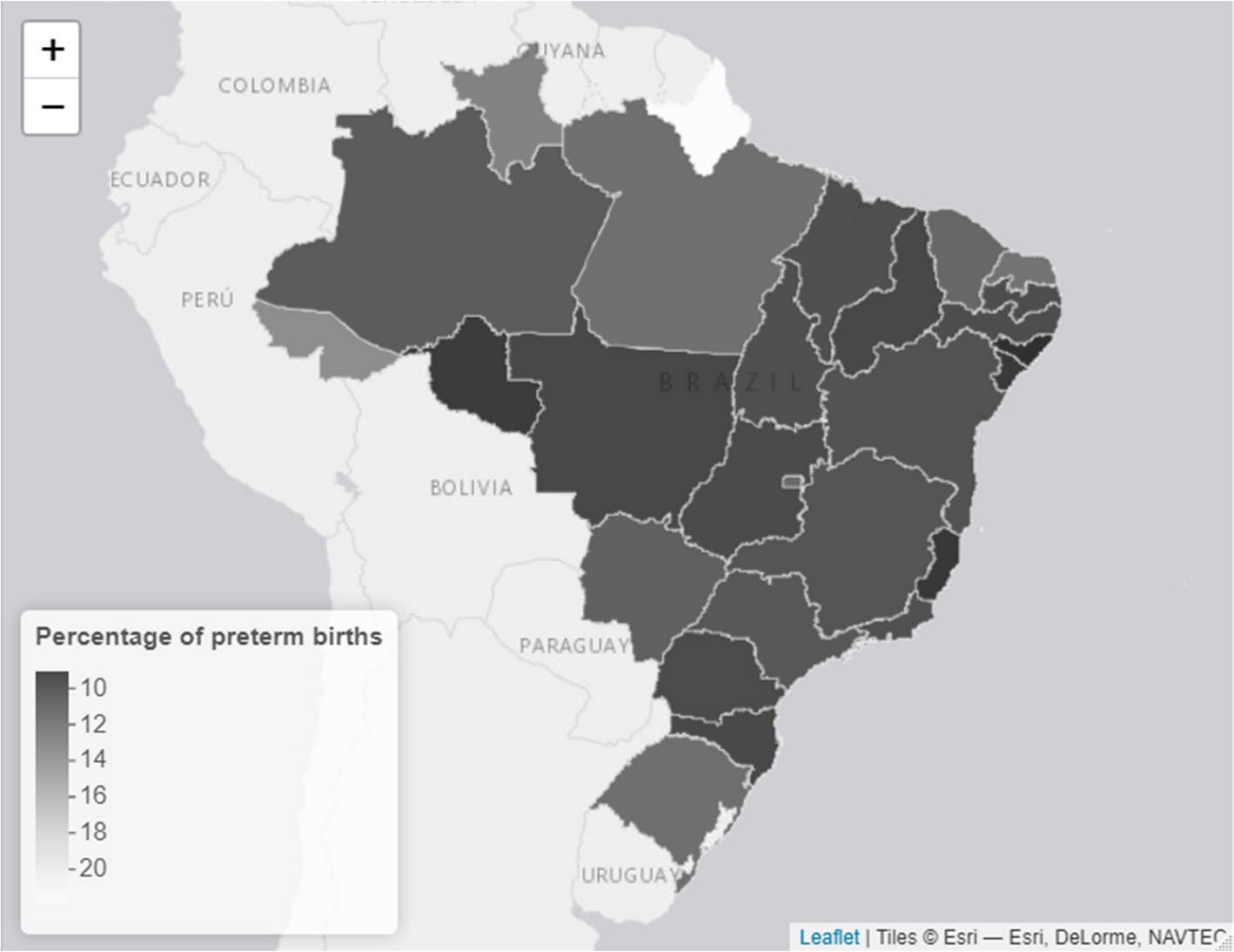
Distribution of Brazilian FU's preterm birth rates in 2019

**Fig. 2:**
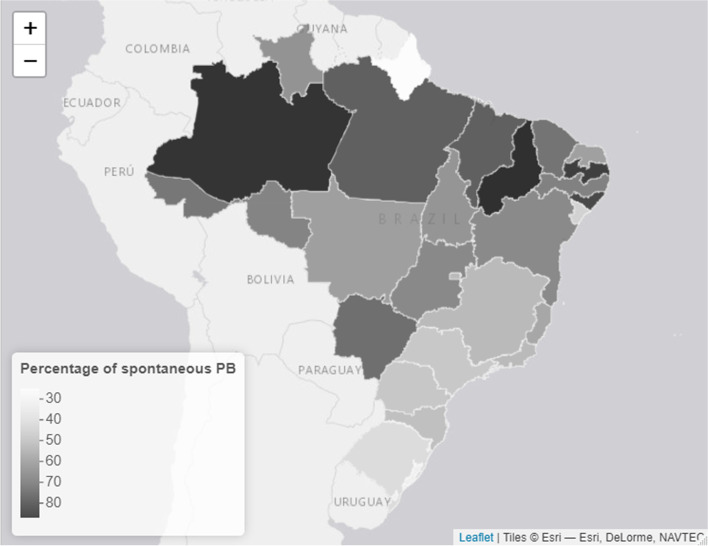
Distribution of Brazilian FU's spontaneous preterm birth fraction in 2019

**Fig. 3:**
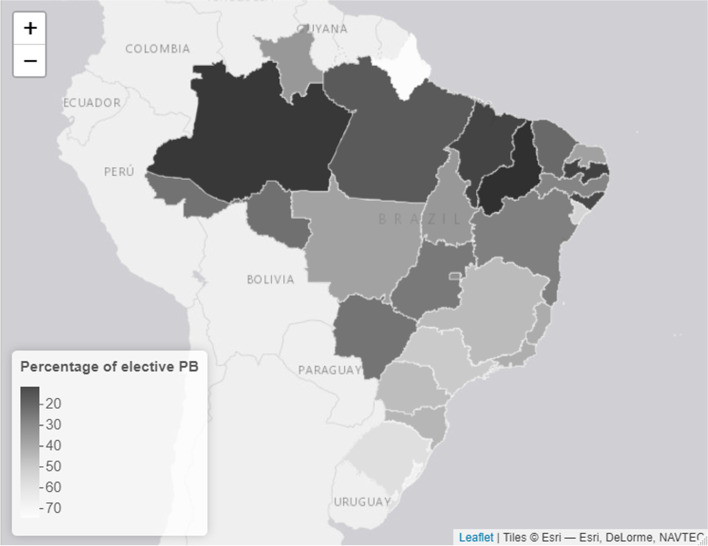
Distribution of Brazilian FU's elective preterm birth fraction in 2019

In 2019, Brazil registered 2,849,146 live births, of which 314,348 (11.03%) were preterm births. The distribution of preterm birth rates throughout the Brazilian FUs in 2019 is illustrated in Fig. 1, and varied from 9.03% in Alagoas to 21.38% in Amapá. Among preterm births, 182,343 and 132,005 were spontaneous and elective births (58% and 42%, constituting 6.39% and 4.63% of total births), respectively. Distribution of spontaneous and elective preterm fractions in FUs are presented in Figs. 2 and 3, respectively.

The highest rate of spontaneous preterm birth was 9.74% in Amazonas, followed by Acre (9.48%). The lowest spontaneous preterm birth rates were 4.2% and 5% in Sergipe and Rio Grande do Sul, respectively. The highest rate of elective preterm birth was recorded in Amapá (16%), followed by Rio Grande do Sul (7%). Detailed data on spontaneous and elective preterm births by FUs are presented in Additional Files 2 and 3, respectively.

### Socioeconomic correlation

#### Total number of preterm births

Spearman's correlation coefficients for the percentage of preterm births with regard to the socioeconomic standards: HDI Education, HDI Longevity, HDI Income, HDI, Gini Index, and life expectancy, were positive (range: *ρ* = 0.03 [HDI Income] to 0.4 [Gini Index]). Thus, higher the values of these variables were associated with a higher percentage of preterm birth (direct relationship). A correlogram of preterm birth rates is presented in Additional File 4.

However, for the rates of illiteracy, access to water, and access to sanitation, we found negative Spearman’s correlation coefficients, which ranged from *ρ* =  − 0.03 (access to sanitation) and *ρ* =  − 0.24 (illiteracy). Thus, the higher the absolute values of the above-mentioned variables, the lower the percentage of preterm births (inverse relationship).

The best fit chosen to model the percentage of preterm birth is shown in Table 1, wherein all variables were significant at the 5% level, and a fixed dispersion model was used.

**Table 1:** Estimated parameters of the final fit to model the rate of preterm births

**Coefficient**	**Estimate**	**Standard error**	**z-value**	***p*-value**
Intercept	− 5.691	0.999	− 5.696	< 0.001
HDI	3.435	1.141	3.011	0.003
Gini Index	2.457	0.716	3.434	< 0.001
Access to sanitation	− 0.006	0.002	− 3.05	0.002
Φ	400.3	108.9	3.674	< 0.001

The expected value of the preterm birth rate as a function of the HDI, Gini Index, and access to sanitation is derived by

$$g({\widehat{\mu }}_{t})=log\left(\frac{{\widehat{\mu }}_{t}}{1-{\widehat{\mu }}_{t}}\right)=-5.691+3.435\times {x}_{1t}+2.457\times {x}_{2t}-0.006\times {x}_{3t}$$

$${\widehat{\mu }}_{t}=\frac{exp(-5.691+3.435\times {x}_{1t}+2.457\times {x}_{2t}-0.006\times {x}_{3t})}{1+exp(-5.691+3.435\times {x}_{1t}+2.457\times {x}_{2t}-0.006\times {x}_{3t})}, t=1,\cdot \cdot \cdot , n,$$

where ${x}_{1}$ represents the HDI, ${x}_{2}$ represents the Gini Index, and ${x}_{3}$ represents access to sanitation.

**Fig. 4:**
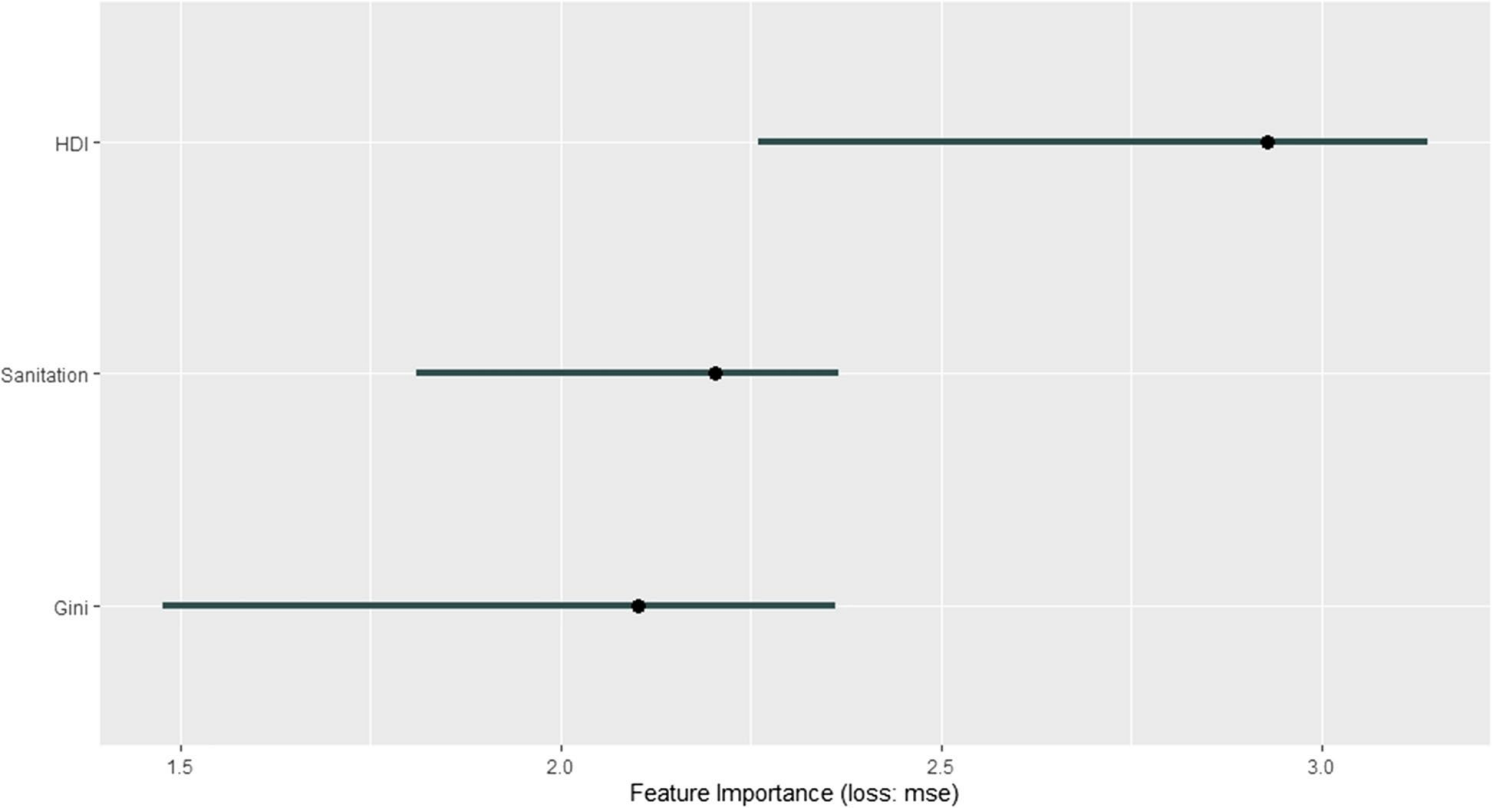
Graph of features importance for the model of the total preterm birth rate

Figure 4 shows the socioeconomic variables importance graph. It is evident that the most important socioeconomic variable is HDI, in which the increase in MSE is about 2.93 with a variability between 2.26 and 3.14. The second most important variable is access to sanitation with the increase in MSE of 2.20 (variability between 1.81 and 2.36) and the less important socioeconomic factor is Gini index (increase in MSE of 2.10 with variability between 1.48 and 2.36).

**Fig. 5:**
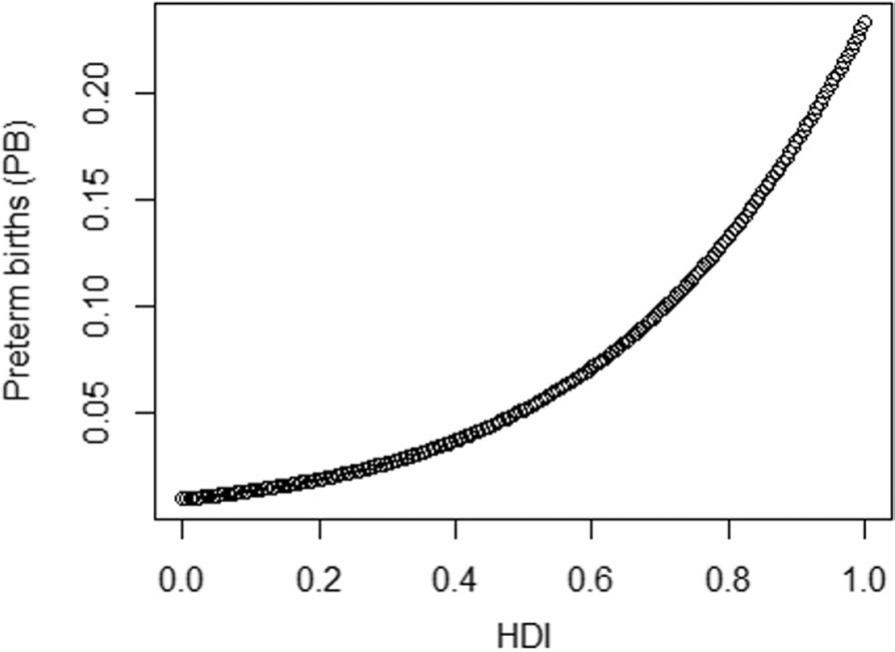
Graph of partial dependence: HDI versus preterm birth when the Gini Index and sanitation are fixed at 0.53 and 39.64%, respectively

For example, a state with an HDI of 0.8, presents an increment of 35.56% in the expected value of the percentage of preterm births compared to a state with an HDI of 0.7, given that the Gini Index and sanitation were fixed at their mean values of 0.53 and 39.64, respectively. Figure 5 shows the partial dependence of the HDI and preterm birth rate, when considering fixed mean values of the Gini Index and sanitation. For the Gini index, a state with a rate of 0.8, for example, has an increment of 22.28% in the estimated expected value of preterm births compared to a state with a Gini Index of 0.7, when HDI and sanitation are fixed at their mean values of 0.75 and 39.64, respectively. Finally, a state with a 50% access to sanitation rate, for example, has a reduction of 5.20% in the estimated expected value of the percentage of preterm births compared to a state with a sanitation rate of 40% when the HDI and Gini Index are fixed at their mean values of 0.75 of 0.53, respectively.

#### Fraction of spontaneous preterm births

Similar results were observed in all three groups (22–27, 28–31, and 32–36 weeks of gestation) of the spontaneous preterm birth fraction; therefore, data are presented by aggregating births from 22 to 36 weeks of gestation.

Spearman's correlation coefficients between the fraction of spontaneous preterm births out of total preterm births and the socioeconomic metrics HDI Education, HDI Longevity, HDI Income, HDI access to water and sanitation, and life expectancy were negative (from *ρ* =  − 0.41 [access to water rate] to *ρ* =  − 0.67 [HDI Income]). Thus, higher values of these variables were associated with a lower percentage of spontaneous preterm births (inverse relationship). The correlogram of the spontaneous preterm birth fraction is presented in Additional File 5.

However, for the Gini Index and the rate of illiteracy, we found positive Spearman correlation coefficients (*ρ* = 0.24 and 0.66, respectively). Thus, higher values the above-mentioned variables corresponded with a higher fraction of spontaneous preterm births (direct relationship).

The best fit chosen to model the spontaneous preterm birth fraction is shown in Table 2, wherein all variables were significant at the 5% level, and a fixed dispersion model was used.

**Table 2:** Estimated parameters of final fit to model percentage of spontaneous preterm birth

**Coefficient**	**Estimate**	**Standard error**	**z-value**
Intercept	6.590	1.752	3.761
HDI	− 8.154	2.326	− 3.506
Φ	15.37	4.064	3.782

All *p* < 0.001

The expected value of the spontaneous preterm birth fraction as a function of the HDI is derived by

$$g({\widehat{\mu }}_{t})=log\left(\frac{{\widehat{\mu }}_{t}}{1-{\widehat{\mu }}_{t}}\right)=6.590-8.154\times {x}_{1t}$$

$${\widehat{\mu }}_{t}=\frac{exp(6.590-8.154\times {x}_{1t})}{1+exp(6.590-8.154\times {x}_{1t})}, t=1,\cdot \cdot \cdot , n,$$

in which ${x}_{1}$ represents the HDI.

**Fig. 6:**
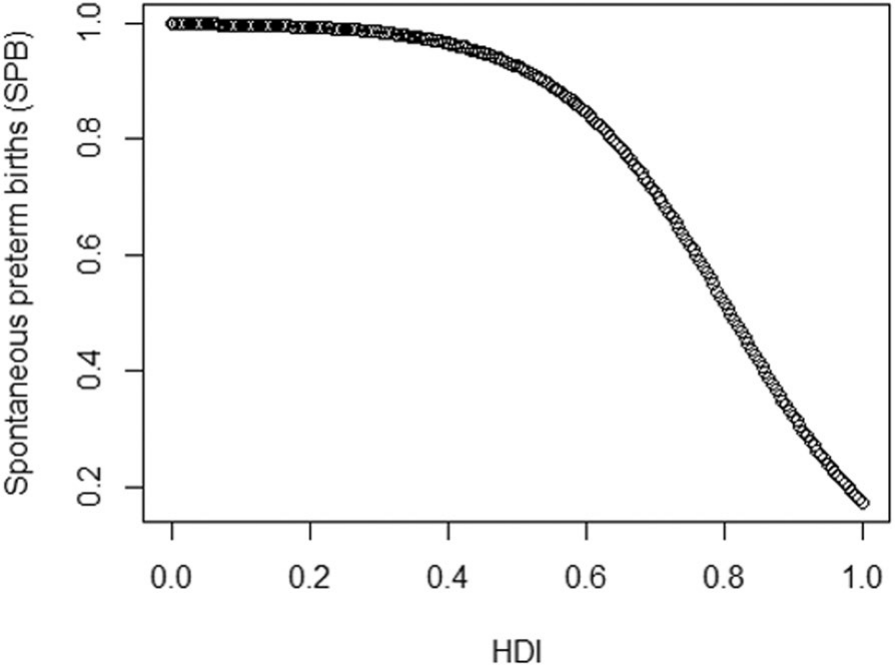
Graph of partial dependence: Human Development Index versus spontaneous preterm births (SPB)

The relationship between the HDI and spontaneous preterm births (Fig. 6) is inverse, which means that as the HDI increases, the fraction of spontaneous preterm births decreases. For example, a state with an HDI of 0.8, for example, presents a reduction of 26.90% in the estimated expected value of the percentage of spontaneous preterm births compared to a state with an HDI of 0.7.

#### Fraction of elective preterm births

Similar results were observed in all three groups of the elective preterm birth fraction (22–27, 28–31, and 32–36 weeks of gestation); therefore, data are presented by aggregating births from 22 to 36 weeks of gestation.

Spearman's correlation coefficients of the fraction of elective preterm births out of the total preterm births with regard to the socioeconomic metrics HDI Education, HDI Longevity, HDI Income, HDI access to water and sanitation, and life expectancy are positive (range from ρ = 0.42 [access to water] to 0.64 [HDI income and HDI]). Thus, a higher value of these variables was associated with a higher elective preterm birth fraction (direct relationship). A correlogram of the elective preterm birth fraction is presented in Additional File 6.

Nevertheless, for the Gini Index and rate of illiteracy, we obtained negative Spearman correlation coefficients (range, ρ =  − 0.28 [Gini Index] and − 0.64 [illiteracy]). Thus, the higher the value of this variable, the lower is the percentage of elective preterm births (inverse relationship).

The best fit chosen to model the elective preterm birth fraction is shown in Table 3, in which all variables were significant at the 5% significance level, and the variable dispersion model was used (conditioning the Gini Index for the parameter of accuracy Φ).

**Table 3:** Estimated parameters of the final fit to model the percentage of elective preterm births

**Coefficient**	**Estimate**	**Standard error**	**z-value**
µ			
Intercept	− 7.994	1.342	− 5.956
HDI	9.795	1.735	5.644
Φ			
Intercept	15.301	3.147	4.862
Gini Index	− 23.065	5.902	− 3.908

All *p* < 0.001

The expected value of the elective preterm birth fraction as a function of the HDI and the Gini Index is given by

$$g({\widehat{\mu }}_{t})=log\left(\frac{{\widehat{\mu }}_{t}}{1-{\widehat{\mu }}_{t}}\right)=-7.994+9.795\times {x}_{1t}$$

$${\widehat{\mu }}_{t}=\frac{exp(-7.994+9.795\times {x}_{1t})}{1+exp(-7.994+9.795\times {x}_{1t})}, t=1,\cdot \cdot \cdot , n,$$

in which ${x}_{1}$ represents the HDI.

**Fig.7:**
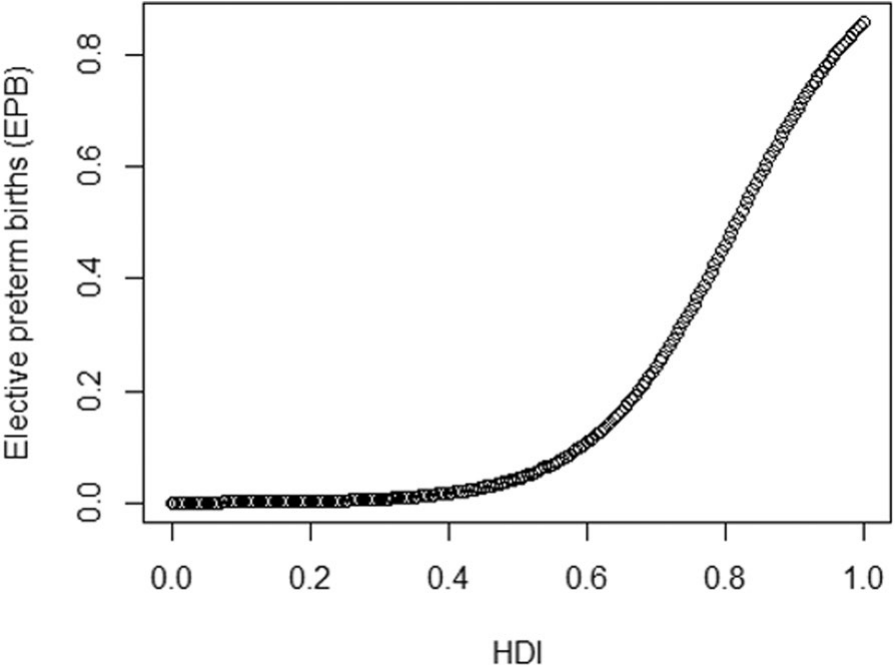
Graph of partial dependence: Human Development Index versus elective preterm birth (EPB)

The relationship between the HDI and the fraction of elective preterm births is direct (Fig. 7). Thus, as the value of the HDI increases, the fraction of elective preterm births also increases. For example, a state with an HDI of 0.8 has an 89.64% increase in the estimated expected value of the percentage of elective preterm births compared to a state with an HDI of 0.7.

## Discussion

To our knowledge, this is the most comprehensive study conducted in developing countries regarding socioeconomic influence on preterm birth rate. We observed that areas with lower levels of development in Brazil were associated with higher spontaneous preterm birth fraction, whereas areas with higher development status were associated with higher fraction of elective preterm birth.

In our study, we found that, in Brazil in 2019, the mean prevalence of preterm births was 11.03% (range, 9.03%–21.38%), which aligns with previous estimates from other studies wherein the prevalence varied from 10.2% to 15% [25, 26]. The last national multicentre study, conducted from April 2011 to July 2012, [27] found a mean prevalence of 12.3%, which is compatible with other recent studies in a FU capital city [28] and with the results of our data analysis. The higher rate of preterm birth in Amapá constitutes an outlier and should be further analysed by temporally comparing the series and consistency of the public register in future years.

With regard to the national fractions of spontaneous and elective preterm births in 2019, 58% were spontaneous, with higher rates in the northern region and lower rates in the south and southeast regions. This finding represents a reduction in the fraction of spontaneous preterm births that infers an increment in the fraction of elective preterm births, compared with the results of a 2014 study, which found that 64.6% of preterm births were spontaneous [27]. Higher rates of spontaneous preterm birth in the north, northeast, and mid-western regions were observed previously [29]. Elevated rates of spontaneous preterm births are associated with regions that have a lower socioeconomic status [30], and a possible proximal explanation may rely on differences in prenatal care coverage [29, 31], which we were unable to verify in the present study.

Considering the total rate of preterm birth, we found a direct relationship between the HDI, the Gini Index, and life expectancy, but discerned an inverse relationship between illiteracy, access to water, and sanitation. Our results present a similar paradox to that observed in other Brazilian studies [28, 32], wherein low birth weight was higher in more developed areas. This could be explained by higher medical assistance and better registration in areas with a higher HDI. Positive association with Gini Index and total preterm births could be explained as a higher burden of spontaneous preterm birth among total births, as well as because of Brazilian elevated indexes of inequality in richer areas.

When analysing spontaneous preterm births, we found a direct relationship between illiteracy and the Gini Index and an inverse relationship between HDI, life expectancy, access to water, and sanitation. Our results indicate that areas with lower levels of education and less equality in income distribution, which are risk factors otherwise proven to be relevant, have a higher fraction of spontaneous preterm birth [31, 33]. Higher HDI and life expectancy, and greater access to water and sanitation all represent higher socioeconomic status that, therefore, predicts a lower spontaneous preterm birth rate, as shown by our data, [30].

Finally, elective preterm birth presented the converse associations of the spontaneous preterm birth analysis. Higher elective preterm birth rates are associated with advances in obstetric procedures and early diagnosis of maternofoetal conditions, all of which occur more often in richer and more developed locations [34]. Increased elective preterm birth rates are commonly accompanied by lower infant mortality and stillbirth rates [34], which indicates better perinatal support. Another important factor is to ascertain whether richer areas may be overproducing preterm births, which could be due to increased access to medical assistance and more incisive surveillance of maternal-foetal conditions.

The main strength of our study was the use of national data, which provided a broad and comprehensive outlook of preterm birth in Brazil. Another unique aspect was the approach adopted to determine both spontaneous and elective fractions of preterm births, which has rarely been explored in Brazilian studies. The use of state-of-the-art statistical methods provides an enriched analysis.

The study’s limitations emerge primarily from the use of public national systems of information. Despite recent enhancements in the quality of SINASC data, underreporting remains an issue [35], especially in less developed areas of the country. Inappropriate records can lead to misleading correlations. Moreover, the lack of more recent census information was detrimental to the accuracy of the analysis, as births in 2019 were compared to socioeconomic indicators, mainly from 2017. Nonetheless, we believe that the impact was minor due to the stability of these metrics over short periods, although this aspect surely limited the interpretation of the results.

## Conclusions

Our study showed that higher socioeconomic status was associated with a higher fraction of elective preterm birth rate and, complementarily, lower spontaneous preterm birth. Preterm birth rate substantially differs depending on the development status of the FU, as well as its distribution in elective and spontaneous births.

The study provides unique and comprehensive continent-sized and population-based data, which can potentially be used as a powerful tool to orient policy decisions aimed at reducing perinatal complications. Orienting our view on preterm birth by two different entities – spontaneous and elective preterm births – allows targeted interventions according to territory socioeconomic parameters aimed at reducing the rate of preterm births and ensuring better outcomes in the morbimortality of infants and mothers.

## Supplementary Information

**Additional file 1.**

**Additional file 2.**

**Additional file 3.**

**Additional file 4.**

**Additional file 5.**

**Additional file 6.**

## Data Availability

The datasets used and/or analysed during the current study are available from the corresponding author upon reasonable request.

## Abbreviations

EPB: Elective preterm birth
Fiocruz: Oswaldo Cruz Foundation
FU: Federal Unit
GNI: Gross national income
HDI: Human Development Index
IBGE: Instituto Brasileiro de Geografia e Estatística [Brazilian Institute of Geography and Statistics
MSE: Mean Square Error
PCDaS: Data Science applied to Health Platform
PROADESS: Health System Performance Assessment Methodology
SINASC: Sistema de Informações sobre Nascidos Vivos [National System of Live Births]
SPB: Spontaneous preterm birth
