# Supplementary material for: Association between preterm births and socioeconomic development: analysis of national data
Source: BMC Public Health. 2022 Nov 3;22:2014. doi: 10.1186/s12889-022-14376-2 (PMC9632029; doi:10.1186/s12889-022-14376-2)

Heatmap of preterm births rates from 2014 to 2019 per Federal Unit of Brazil.

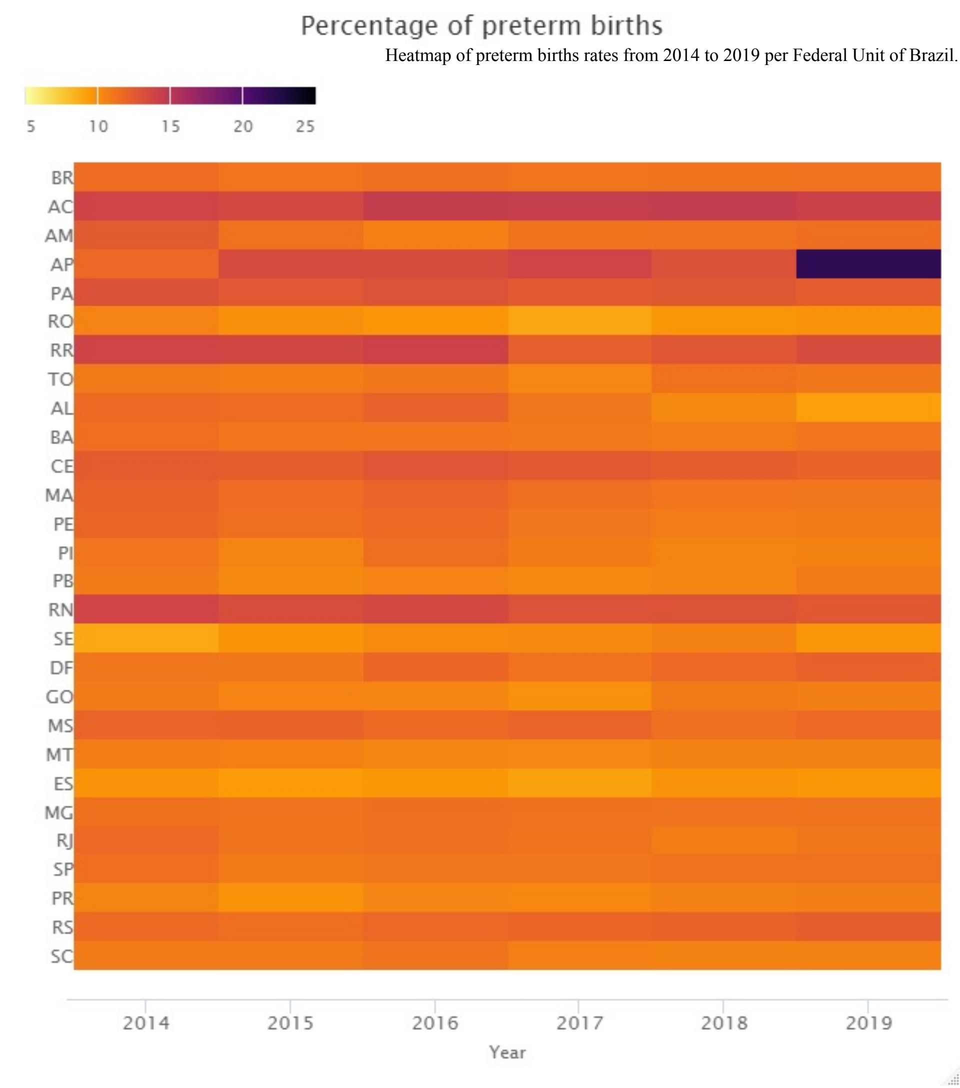

Supplement: Supplementary file 1 — Additional file 1. [file 12889_2022_14376_MOESM1_ESM.pdf]
