# Supplementary material for: Association between preterm births and socioeconomic development: analysis of national data
Source: BMC Public Health. 2022 Nov 3;22:2014. doi: 10.1186/s12889-022-14376-2 (PMC9632029; doi:10.1186/s12889-022-14376-2)
Supplement: Supplementary file 2 — Additional file 2. [file 12889_2022_14376_MOESM2_ESM.pdf]

Table of spontaneous preterm births per Federal Unit of Brazil

FU	*Total births*	*PB*	*Spontaneous PB*
DF	42422	5055 (11.92%)	2596 (6.12%)
SP	583191	65403 (11.21%)	31291 (5.37%)
SC	98032	10213 (10.42%)	5096 (5.20%)
RJ	207989	22510 (10.82%)	11524 (5.54%)
PR	153469	16193 (10.55%)	7791 (5.08%)
MG	256892	28259 (11.00%)	14504 (5.65%)
RS	134596	16425 (12.20%)	6731 (5.00%)
MT	58852	6115 (10.39%)	3562 (6.05%)
ES	54925	5199 (9.47%)	2910 (5.30%)
MS	43695	5087 (11.64%)	3638 (8.33%)
RR	14620	1953 (13.36%)	1202 (8.22%)
TO	24449	2642 (10.81%)	1608 (6.58%)
AP	15356	3283 (21.38%)	826 (5.38%)
CE	129185	14872 (11.51%)	10480 (8.11%)
AM	77622	8832 (11.38%)	7558 (9.74%)
RN	44031	5434 (12.34%)	3136 (7.12%)
PE	133359	14049 (10.53%)	9338 (7.00%)
RO	27028	2500 (9.25%)	1639 (6.06%)
PB	57701	6143 (10.65%)	5116 (8.87%)
AC	16280	2221 (13.64%)	1543 (9.48%)
BA	197249	21025 (10.66%)	13443 (6.82%)
SE	32697	3101 (9.48%)	1373 (4.20%)

PA	138341	16247 (11.74%)	12101 (8.75%)
PI	47933	4915 (10.25%)	4247 (8.86%)
MA	113317	11991 (10.58%)	8955 (7.90%)
AL	49803	4495 (9.03%)	3598 (7.22%)
Total	2849146	314348 (11.03%)	182343 (6.40%)
