# Supplementary material for: Association between preterm births and socioeconomic development: analysis of national data
Source: BMC Public Health. 2022 Nov 3;22:2014. doi: 10.1186/s12889-022-14376-2 (PMC9632029; doi:10.1186/s12889-022-14376-2)
Supplement: Supplementary file 3 — Additional file 3. [file 12889_2022_14376_MOESM3_ESM.pdf]

Table of elective preterm births per Federal Unit of Brazil.

FU	Total births	PB	Elective PB
DF	42422	5055 (11.92%)	2459 (5.80%)
SP	583191	65403 (11.21%)	34112 (5.85%)
SC	98032	10213 (10.42%)	5117 (5.22%)
RJ	207989	22510 (10.82%)	10986 (5.28%)
PR	153469	16193 (10.55%)	8402 (5.47%)
MG	256892	28259 (11.00%)	13755 (5.35%)
RS	134596	16425 (12.20%)	9694 (7.20%)
MT	58852	6115 (10.39%)	2553 (4.34%)
ES	54925	5199 (9.47%)	2289 (4.17%)
GO	96112	10186 (10.60%)	3649 (3.80%)
MS	43695	5087 (11.64%)	1449 (3.32%)
RR	14620	1953 (13.36%)	751 (5.14%)
TO	24449	2642 (10.81%)	1034 (4.23%)
AP	15356	3283 (21.38%)	2457 (16.00%)
CE	129185	14872 (11.51%)	4392 (3.40%)
AM	77622	8832 (11.38%)	1274 (1.64%)
RN	44031	5434 (12.34%)	2298 (5.22%)
PE	133359	14049 (10.53%)	4711 (3.53%)
RO	27028	2500 (9.25%)	861 (3.19%)
PB	57701	6143 (10.65%)	1027 (1.78%)
AC	16280	2221 (13.64%)	678 (4.16%)
BA	197249	21025 (10.66%)	7582 (3.84%)
SE	32697	3101 (9.48%)	1728 (5.28%)

PA	138341	16247 (11.74%)	4146 (3.00%)
PI	47933	4915 (10.25%)	668 (1.39%)
MA	113317	11991 (10.58%)	3036 (2.68%)
AL	49803	4495 (9.03%)	897 (1.80%)
Total	2849146	314348 (11.03%)	132005 (4.63%)
