# Supplementary figures and images for: Association between preterm births and socioeconomic development: analysis of national data
Source: BMC Public Health. 2022 Nov 3;22:2014. doi: 10.1186/s12889-022-14376-2 (PMC9632029; doi:10.1186/s12889-022-14376-2)

Correlogram of preterm births rates from 2019.

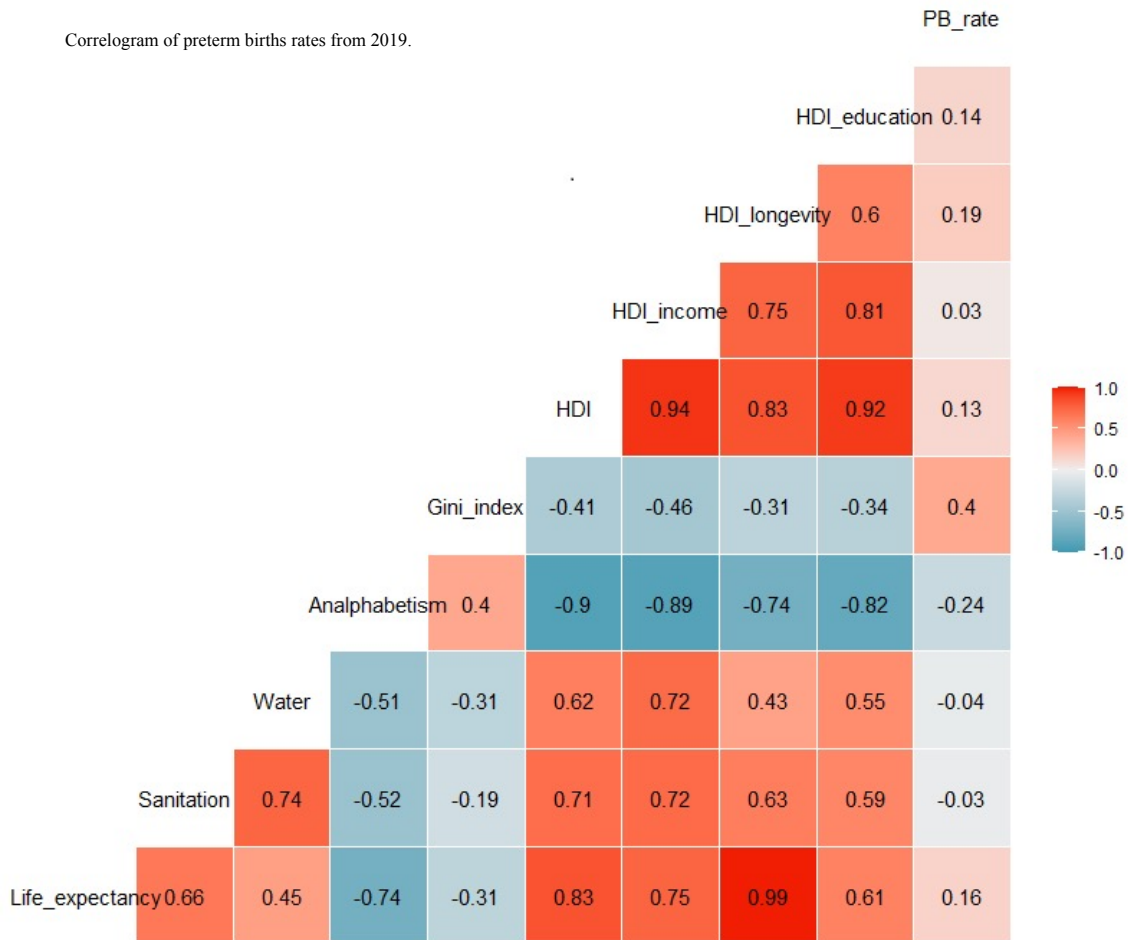

Supplement: Supplementary file 4 — Additional file 4. [file 12889_2022_14376_MOESM4_ESM.pdf]

SPB\_rate

Correlogram of spontaneous fraction of preterm births from 2019.

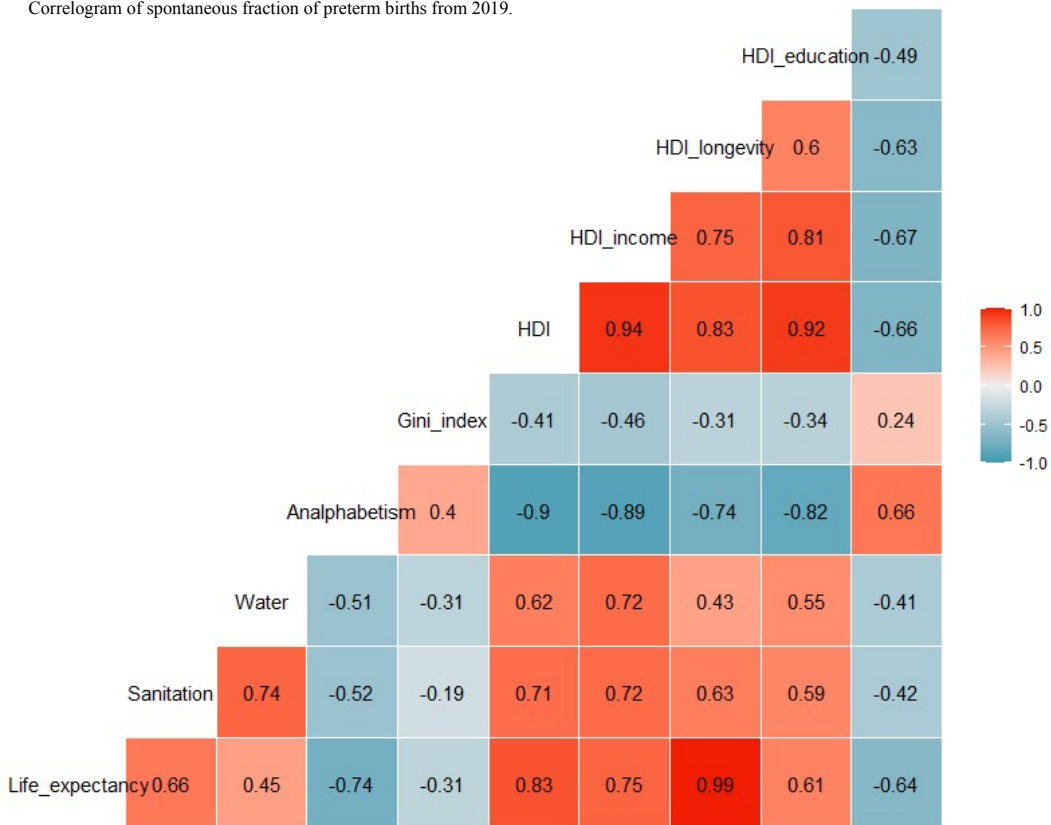

Supplement: Supplementary file 5 — Additional file 5. [file 12889_2022_14376_MOESM5_ESM.pdf]

Correlogram of elective fraction of preterm births from 2019.

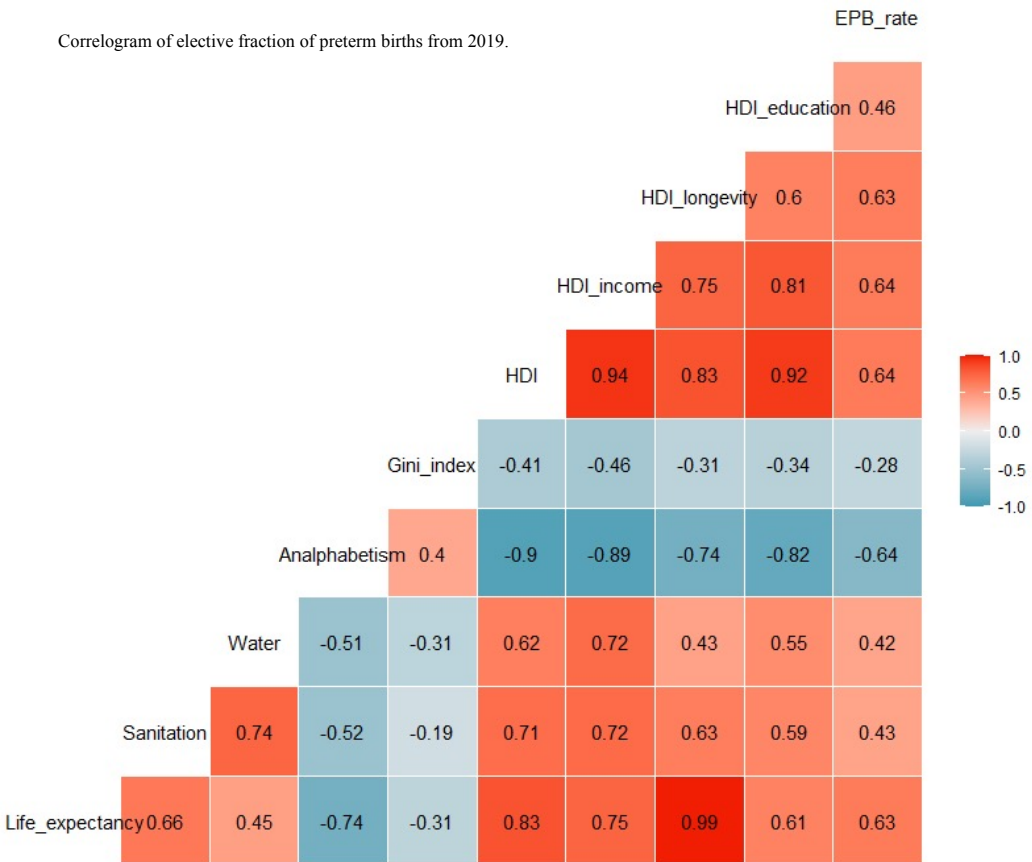

Supplement: Supplementary file 6 — Additional file 6. [file 12889_2022_14376_MOESM6_ESM.pdf]
